# Small and genetically highly structured populations in a long-legged bee, *Rediviva longimanus*, as inferred by pooled RAD-seq

**DOI:** 10.1186/s12862-018-1313-z

**Published:** 2018-12-19

**Authors:** Belinda Kahnt, Panagiotis Theodorou, Antonella Soro, Hilke Hollens-Kuhr, Michael Kuhlmann, Anton Pauw, Robert J. Paxton

**Affiliations:** 1 General Zoology, Institute of Biology, Martin-Luther-University Halle-Wittenberg, Hoher Weg 8, 06120 Halle (Saale), Germany; 2 German Centre for Integrative Biodiversity Research (iDiv) Halle-Jena-Leipzig, Deutscher Platz 5e, 04103 Leipzig, Germany; 3 Institute of Landscape Ecology, Westfälische Wilhelms-Universität Münster, Heisenbergstraße 2, 48149 Münster, Germany; 4 Zoological Museum, Kiel University, Hegewischstr. 3, 24105 Kiel, Germany; 5 Department of Life Sciences, Natural History Museum, Cromwell Road, London, SW7 5BD UK; 6 Department of Botany and Zoology, Stellenbosch University, Matieland, 7602 South Africa

**Keywords:** Population genomics, Population genetic structure, Pollinators, Pool-Seq, Ecological adaptation, South Africa, Selection

## Abstract

**Abstract:**

Adaptation to local host plants may impact a pollinator’s population genetic structure by reducing gene flow and driving population genetic differentiation, representing an early stage of ecological speciation. South African *Rediviva longimanus* bees exhibit elongated forelegs, a bizarre adaptation for collecting oil from floral spurs of their *Diascia* hosts. Furthermore, *R. longimanus* foreleg length (FLL) differs significantly among populations, which has been hypothesised to result from selection imposed by inter-population variation in *Diascia* floral spur length. Here, we used a pooled restriction site-associated DNA sequencing (pooled RAD-seq) approach to investigate the population genetic structure of *R. longimanus* and to test if phenotypic differences in FLL translate into increased genetic differentiation (i) between *R. longimanus* populations and (ii) between phenotypes across populations. We also inferred the effects of demographic processes on population genetic structure and tested for genetic markers underpinning local adaptation.

**Results:**

Populations showed marked genetic differentiation (average *F*_ST_ = 0.165), though differentiation was not statistically associated with differences between populations in FLL. All populations exhibited very low genetic diversity and were inferred to have gone through recent bottleneck events, suggesting extremely low effective population sizes. Genetic differentiation between samples pooled by leg length (short versus long) rather than by population of origin was even higher (*F*_ST_ = 0.260) than between populations, suggesting reduced interbreeding between long and short-legged individuals. Signatures of selection were detected in 1119 (3.8%) of a total of 29,721 SNP markers,

**Conclusions:**

Populations of *R. longimanus* appear to be small, bottlenecked and isolated. Though we could not detect the effect of local adaptation (FLL in response to floral spurs of host plants) on population genetic differentiation, short and long legged bees appeared to be partially differentiated, suggesting incipient ecological speciation. To test this hypothesis, greater resolution through the use of individual-based whole-genome analyses is now needed to quantify the degree of reproductive isolation between long and short legged bees between and even within populations.

**Electronic supplementary material:**

The online version of this article (10.1186/s12862-018-1313-z) contains supplementary material, which is available to authorized users.

## Background

Mating usually takes place among a subset of individuals of a species, typically within a portion of its distributional range, and, assuming limited dispersal, populations of that species inevitably become genetically structured [1]. The population genetic structure of populations is then determined in part by the strength of two parameters: effective population size (*N*_*e*_) and the amount of gene flow among populations [1]. Low *N*_*e*_ is a typical feature of rare or endangered species [2] or of populations that have experienced a recent bottleneck, such as founder populations [1]. Limited gene flow may be caused by fragmentation of the landscape due to human interferences (e.g. agriculture, transport), natural barriers (e.g. waterways, mountains) or abiotic factors (e.g. climate). The environment might also exert non-negligible selection pressures upon populations, whereby adaption to ecologically different environments might reduce intraspecific gene flow and lead to reproductive barriers (e.g. selection against hybrids or immigrants, positive assortative mating) between populations or individuals varying in adaptive traits [3–6], which may represent incipient stages of ecological speciation [7].

Host plant adaptation seems to be a common feature of many insect-plant interaction systems [5, 8–13]. Adaptation to different host plant morphologies might hinder genetic exchange between insect populations and generate strong barriers to gene flow [14]. Such populations may then accumulate allele frequency differences, i.e. become genetically differentiated, often assessed via *F*_ST,_ which relates the amount of genetic variation among populations to the total genetic variation over all populations [15]. Initially, increased genetic differentiation is expected at loci underlying local adaptation whereas ecologically neutral loci are not subject to divergent selection and should therefore show less differentiation. This generates a heterogeneous pattern of genomic divergence characterised by ‘islands of genomic divergence’ containing *F*_ST_ outlier loci [16, 17]. The more adaptively divergent populations become, the greater the reduction in gene flow and the higher the genome-wide differentiation, yielding a pattern of isolation by adaptation (IBA, [18]). Host plant mediated genetic differentiation and incipient ecological speciation have been suggested for several insects [4, 5, 9, 19, 20].

South African *Rediviva* bees are striking examples of the bizarre morphology that host plant adaptation may generate. Females of many *Rediviva* species have evolved extremely elongated forelegs, sometimes longer than their entire body length [21, 22]. Forelegs are used for oil collection from oil-producing plants, whereby the bee inserts its forelegs into the host floral spurs and rubs them against the spur walls to absorb oil with specialised hairs on the tarsi [23, 24]. The extracted oil is then transported back to the nest and used to feed larvae and probably also for brood cell lining [25, 26].

Foreleg length (FLL) of *Rediviva* females is an evolutionary highly labile trait which likely plays a role in *Rediviva* diversification [27]. Moreover, FLL of *Rediviva* varies not only between species but also between populations of the same species [21, 24, 28, 29], in which intraspecific variation in FLL has been shown to correlate with floral spur length of the main host plant *Diascia* [28, 29]*.* Since most *Rediviva* use a range of *Diascia* [21, 22] or other plant taxa (other Scrophulariaceae, Orchidaeceae, Iridaceae, Stilbaceae) as sources of oil [24, 26, 30–32], FLL might evolve in response to the spur length of the local community rather than to an individual host plant species, ([33], Hollens-Kuhr et al., unpublished observations), i.e. diffuse coevolution. A close match between *Rediviva* FLL and host plant spur lengths is, however, still necessary for successful oil extraction as the main host, *Diascia,* only produces oil in the distal end of the spurs (but see [34]) and thus only bees with sufficiently long forelegs are able to gather oil [24, 34]. Hence, FLL might experience strong selection to match the main host plant’s spur length.

Other factors may impact the genetic structure of *Rediviva* spp. populations beyond adaptation to host flower spur length. The majority of *Rediviva* species occur in the winter-rainfall area of South Africa [21, 22, 35], termed the Succulent Karoo biodiversity hotspot [36], which is characterised by ≥50% of the annual precipitation falling during winter [37]. Predominantly cold, rainy, and cloudy conditions during the main flowering season force winter-active bees, such as most *Rediviva* spp., to concentrate their foraging and brood cell provisioning activities to the short interludes of favourable weather. Hence, bees in this area likely have reduced daily activity and limited dispersal [36], which might reduce gene flow and increase genetic differentiation among populations. Furthermore, as *Rediviva* bees are thought to have special nesting requirements [38], regions of unsuitable habitat might further isolate *Rediviva* populations and reduce gene flow, as hypothesised for other ground-nesting bees in this area [39]. For example, *Rediviva intermixta* prefers loamy dolerite soil [25] whereas *Rediviva peringueyi* is unable to nest in unconsolidated, sandy soil [38]. In addition to the potential reduction in gene flow, some *Rediviva* bee species are probably characterised by a relatively low *N*_*e*_ since they occur in small and scattered populations (Kuhlmann, Hollens-Kuhr, unpublished observations).

Here, we used a restriction site-associated DNA sequencing (RAD-seq) approach to investigate the population genetic structure and demography of *Rediviva longimanus.* Specifically, in a population-based pooled RAD-seq dataset, we tested whether phenotypic differentiation in FLL translates into increased genetic differentiation between populations (isolation by adaptation: IBA) over the purely neutral evolutionary process of genetic drift (isolation by distance: IBD). *Rediviva longimanus* is among the *Rediviva* species (FLL = 6–23 mm) with the most extreme FLL (x̄=21 mm) and populations show noticeable differences in FLL, even over a small geographic scale [21], rendering it a particularly suitable study system with which to test for reproductive isolation related to local adaptation. We also measured differentiation between long-legged and short-legged bees within and between populations in a second pooled RAD-seq dataset, representing another test of incipient ecological speciation. We finally used an empirical *F*_ST_-outlier approach as well as a PCA-based outlier detection test to identify loci underpinning local adaptation.

## Methods

### Study species and sampling sites

**Fig. 1:**
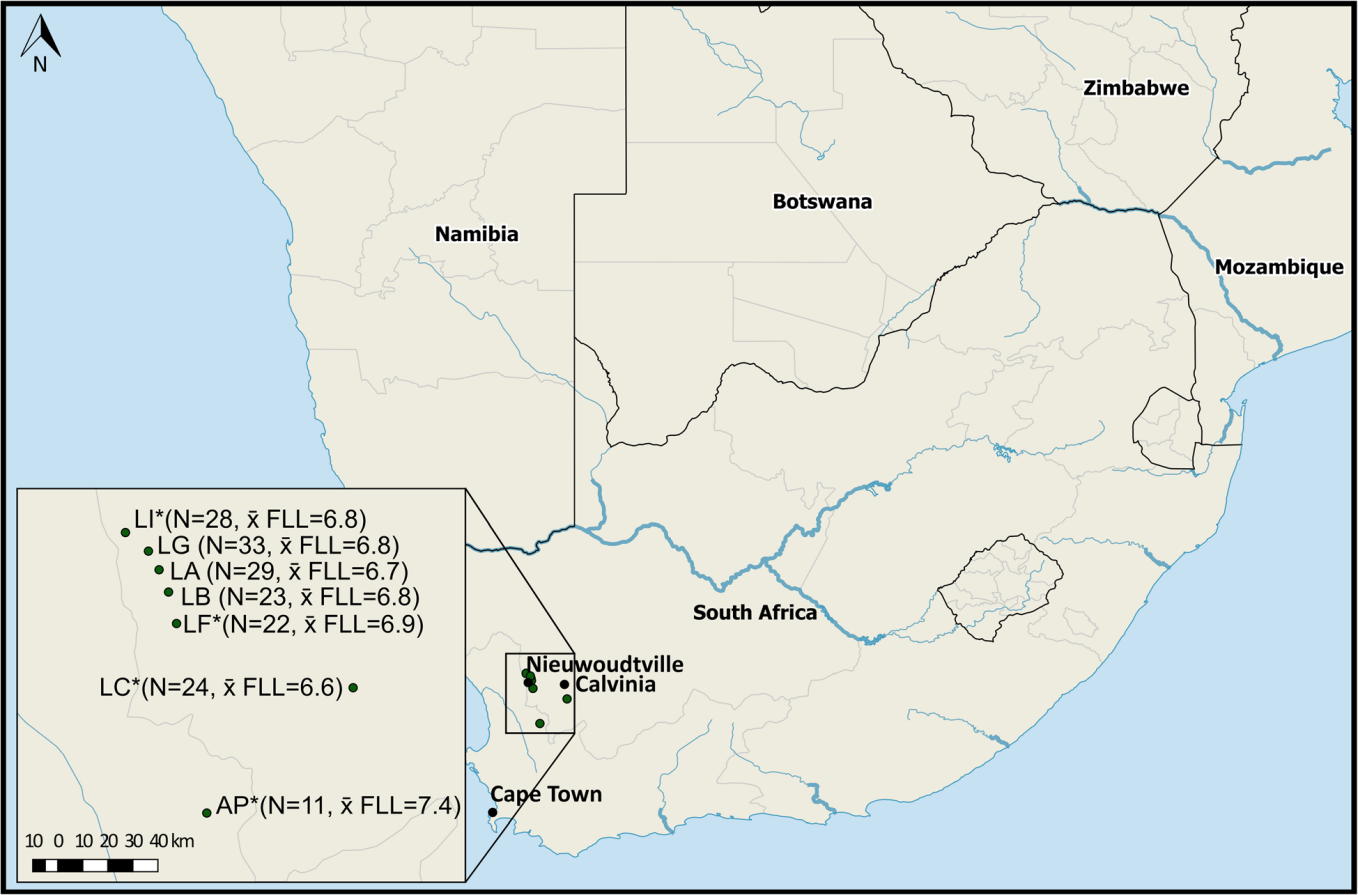
Sampling locations of *R. longimanus* populations in South Africa. Population labels with an asterisk correspond to the four *population pools* (AP, LC, LI, LF) used for population genetic analysis in this study. For the two *leg pools*, we included individuals from LC, LI and LF as well as from three other populations (LA, LB, LG) to obtain two pools comprising either individuals with the longest or the shortest foreleg length (FLL), irrespective of population of origin. Sample sizes and mean relative FLL are given in brackets

*Rediviva longimanus* is endemic to the Succulent Karoo in Western South Africa. Its distribution encompasses the Cederberg Mountains in the west, the Roggeveld Mountains in the east and the Nieuwoudtville area in the north [21]. Sampling of female bees was conducted at seven sites located near the towns of Nieuwoudtville, Calvinia and Clanwilliam (Fig. 1, Table 1), where *R. longimanus*, though rare, is abundant enough to be sampled and where we expected to find differences in FLL even across a small geographic scale (Hollens-Kuhr et al., unpublished observations).

**Table 1:** Sampling locations of *Rediviva longimanus* populations

Code	Location	Latitude	Longitude	N	x̄ abs. FLL	SD	x̄ rel. FLL	SD
AP*	Biedouw Valley	32° 14′ 76.7”	19°18′ 47.0”	11	20.62	0.86	7.36	0.20
LC*	Keiskie-Mountain	31° 45′ 45.6”	19° 50′ 21.4”	24	18.02	0.52	6.56	0.20
LF*	Farm Papkuilsfontain	31° 33′ 32.0’	19° 10′ 46.5”	22	18.95	0.43	6.87	0.25
LI*	Farm Avontuur	31° 16′ 14.3”	19° 02′ 53.9”	28	18.82	0.63	6.80	0.22
LA	Flower Reserve	31° 21′ 55.9”	19° 08′ 34.9”	29	18.45	0.52	6.73	0.18
LB	Hantam Botanical Garden	31° 24′ 23.7”	19° 09′ 03.8”	23	18.81	0.50	6.81	0.18
LG	Nieuwoudtville Waterfall	31° 19′ 28.1’	19° 07′ 50.8”	33	18.55	0.61	6.78	0.19

Population labels indicated with an asterisk correspond to the *population pools* used for analyses of population genetic structure and FLL outlier identification. Samples from these and additional populations were used to generate the *leg pools* to test for differentiation with respect to leg morphology as well as to identify candidate loci for leg length. For each sampled population, the geographic coordinates (latitude and longitude), sample size (N of females) and the mean absolute (x̄ abs. FLL) and relative (x̄ rel. FLL, see text for definition of relative) FLL with their corresponding standard deviations (SD) are given

### DNA extraction and RAD-seq

DNA was extracted from the thorax, legs or head of females using a DTAB protocol (modified from [40]), which consists of a digestion step with proteinase K in DTAB buffer, followed by extraction with chloroform:isoamyl alcohol 24:1. DNA quality and quantity were assessed using an Epoch spectrophotometer (BioTek, Winooski, USA), by agarose gel electrophoresis and with a Qubit 3.0 fluorometer (Thermo Fisher Scientific, Waltham, USA). Only non-degraded and intact DNA samples were further processed. We first DNA barcoded each individual bee by sequencing the mitochondrial *cytochrome oxidase I* ‘animal barcode’ region [41] and were able to confirm species identity, i.e. *Rediviva longimanus*, for each sample included.

We then pooled individual DNA extracts according to two pooling schemes for restriction site-associated DNA sequencing (RAD-seq). In order to infer the population genetic structure and demography of *R. longimanus* populations, we pooled individuals into four *population pools* corresponding to four of seven sampling locations (Fig. 1, Table 1): AP (*N* = 11), LC (*N* = 24), LF (*N* = 22), LI (*N* = 28). These sites encompassed the range of FLL in *R. longimanus* and differed significantly in mean relative FLL (except LI versus LF, see Additional files 1 and 2), calculated as in [27], i.e. foreleg length divided by head width. We decided to use relative rather than absolute FLL to account for variation in FLL that might be due to variation in overall body size. We note, however, that head width, a proxy for body size [42], varied little between individuals and results of our study (e.g. multiple matrix regression, see below) did not change qualitatively when using absolute rather than relative FLL (data not shown).

In the second pooling scheme, we used samples from six of seven sites to generate two pools according to FLL, to which we refer as *leg pools* (Table 1, Fig. 1, see Additional file 3). One pool consisted of the twenty *R. longimanus* individuals with the overall longest relative FLL and the other pool consisted of twenty individuals showing the shortest relative FLL. Mean relative FLL of the long *leg pool* (x̄ = 7.1 ± 0.16 SD) was significantly different (LM, two-tailed test, *t* = − 14.2, *P* < 0.01) from the short *leg pool* (x̄=6.4 ± 0.13 SD).

In the *leg pools*, we pooled bees showing the most extreme foreleg lengths, i.e. shortest and longest, independent of their population of origin, because we were interested in testing for genetic differentiation with respect to FLL across populations, which might be indicative of the initial stage of ecological speciation. Pooling individuals from different populations but with the same morphology, as in our two *leg pools*, has been recognised as a valid and highly useful approach, especially for the identification of candidate genes for host adaptation [4, 5]. Although each of our *leg pools* comprised individuals from five of seven populations (Additional file 2), we lacked sufficient long or short legged bees from some populations to allow a balanced sampling design. A caveat of our approach, then, is that a component of the genetic differentiation we detected among the *leg pools* dataset might be due to population differentiation.

For each pool, 1.5 μg of genomic DNA, normalised to a final DNA concentration of 60 ng/μl, was sent to Floragenex, Inc. (Eugene, USA) for RAD-seq. RAD-seq is an increasingly used [43–46] *Next Generation Sequencing* approach that yields a reduced representation of the whole genome. By using restriction enzymes that cut DNA at restriction enzyme-specific sites, which occur randomly over the genome, one obtains DNA fragments that are sheared to generate sizes appropriate for sequencing [47]. Subsequent sequencing of homologues fragments in several individuals or pools is able to reveal thousands of single nucleotide polymorphisms (SNPs), which can be analysed in a population genomic framework [47]. RAD-seq was carried out according to the original RAD-seq protocol [47, 48]. DNA was digested using the restriction enzyme *PstI*, randomly sheared and adapters with unique multiplex identifier (MID) ‘barcodes’ (10 bp) for each pool sample were attached to the DNA fragments prior to sequencing. Pooled libraries were run on an Illumina HiSeq2500 platform (Illumina, San Diego, USA) to generate 125 bp single-end reads.

### Data processing and SNP calling

Following a quality control in FASTQC v. 0.11.5 [49], sequence reads were demultiplexed, filtered for quality and trimmed of 10 bp MID sequences using STACKS v. 1.42 [50] under default settings. Since there is no reference genome available for *R. longimanus* or a close relative, we identified RAD loci de novo using *denovo_map.pl* in STACKS (m = 5, M = 2, *n* = 0). Moreover, we remove highly repetitive stacks, loci with a log-likelihood below − 20 and confounding loci, i.e. multiple genomic loci matching a single catalogue locus.

Since STACKS was not specifically designed for the use of data from pooled samples and its SNP calling algorithm is therefore likely to miss low frequency variants in the pool, we used POPOOLATION2 [51] for SNP calling. We mapped all our RAD reads against the reference catalogue created in STACKS using the *bwa mem* algorithm of BWA v. 0.7.12 [52]. Mapping results were filtered for a minimum Phred quality score of 20 and converted into mpileup format in SAMTOOLS v. 0.1.19 [53]. For each population pair, we then calculated the allele frequency difference at each position with a minimum coverage of 10 and a minimum minor allele count of 2 using the *snp-frequency-diff.pl* script of POPOOLATION2 (also see Additional file 4).

### Genome-wide variation and population genetic structure

Genome-wide patterns of genetic diversity were assessed by calculating the population mutation rate (Watterson’s *θ*) and nucleotide diversity (Tajima’s *Π*) in NPSTAT v.1.0 [54]. The accuracy of allele frequency estimates in pooled samples can be increased not only by high sequence coverage but also by using large sliding windows as this avoids incorrect estimates due to stochastic error [55]. To do so, we concatenated all RAD tags and calculated genetic diversity measures over this continuous sequence stretch (one window) for positions with a minimum coverage of 10, minimum count of the minor allele of 2 and a minimum Phred score of 20.

Pairwise and overall genetic differentiation were estimated as the fixation index *F*_ST_ [1] in POPOOLATION2, only considering positions with a minimum coverage of ten and a minimum minor allele count of two per RAD locus i.e. using windows of 115 bp (125 bp reads minus 10 bp MID). However, we also checked the robustness of our estimates by repeating the calculations under even more conservative settings (minimum coverage = 20, minimum minor allele count = 6); results did not qualitatively change. In order to exclude repetitive regions, we set a maximum coverage threshold to exclude those loci with the 2% highest coverage (> 73x for AP, > 58x for LC, > 60x for LF, > 51x for LI) from genetic diversity and *F*_ST_ calculations. All other parameters were left as default. In addition, we recalculated *F*_ST_ after removing loci potentially under selection, i.e. loci identified in either PCADAPT or the tails of the *F*_ST_ distribution (see below), to account for a potential bias in our *F*_ST_ estimates due to selection. Confidence intervals (CI’s) for the *F*_ST_ estimates were inferred by bootstrapping 1000 times in the *R* package BOOTSTRAP v. 2017.2 [56]. In addition to *F*_ST_, we also assessed population genetic structure by principal component analysis (PCA) in the *R* package PCADAPT v. 3.0.4 [57]*.*

We then investigated if population genetic differentiation in the *population pool*s could be explained by differences in FLL (isolation by adaptation, IBA) or geographic distance (isolation by distance, IBD). We regressed the matrices of pairwise population genetic differentiation, transformed to *F*_ST_/(1-*F*_ST_), on relative FLL and on log-transformed geographic distances using a multiple matrix regression with randomisation (MMRR) analysis [58] with 999 permutations in the *R* package ECODIST v. 1.2.2 [59] to avoid pseudoreplication because of the non-independence of *F*_*ST*_ values within a dataset. Geographic distances between population pairs were inferred via the shortest path in GOOGLE EARTH v. 6.2 (Table 3).

### Demographic history of *Rediviva longimanus*

Since estimates for Watterson’s *θ* and Tajima’s *Π* suggested very low genomic diversity for all *population pools* (see Results below), we tested for a bottleneck in each population using FASTSIMCOAL2 v. 2.5.2.21 [60]. We first excluded RAD tags with SNPs potentially under selection (see below) using a custom bash script, and then produced the folded (i.e. based on the allele frequencies of only the minor allele) site frequency spectrum (SFS) in POOL-HMM v. 1.4.3 [61].

In FASTSIMCOAL2 we first estimated model parameters using sequential Markov coalescence simulations and a conditional maximization algorithm (ECM, [60]). In addition to a bottleneck scenario, we also modelled a constant population size scenario and a population expansion scenario. Model comparisons were performed according to the Akaike Information Criterion (*AIC*) and Akaike’s weight of evidence (*w*), as suggested by Excoffier et al. ([59], see Additional file 4 for more details).

### Genetic differentiation by FLL using *leg pools*

We also computed *F*_ST_ between the *leg pools* dataset to measure the effect of FLL on genetic differentiation and to infer potential reproductive isolation due to FLL per se. *F*_ST_ computations in POPOOLATION2 were carried out using the same settings as for the *population pools* (minimum coverage = 10, minimum minor allele count = 2, loci with the 2% highest coverage excluded). *F*_ST_ calculations were repeated after excluding potential outlier loci, i.e. in the 5% tails of the *F*_ST_ distribution (see below). Bootstrapping was performed with 1000 replicates to generate CI’s for the *F*_ST_ estimate.

### Outlier SNP detection

We tested for signals of selection using two outlier detection approaches with both *population pools* and *leg pools* datasets. In the first approach we extracted loci in the lower and upper tails (0.5% for the *population pools* and 5% for the *leg pools*) of the *F*_ST_ distribution, as calculated in POPOOLATION2 (also see Additional file 4). The *F*_ST_ outlier criterion of 5% for the *leg pools* differed from that for the *population pools* because it already incorporated the maximum value of *F*_ST_ = 1. We considered the loci with the highest *F*_ST_ values (upper tail) as candidates for divergent selection and the loci with the lowest *F*_ST_ values (lower tail) as candidates for balancing selection, in accordance with the rationale underlying *F*_ST_-outlier detection tools such as BAYESCAN [62] or LOSITAN [63].

In the second approach to infer signals of selection, we used PCADAPT v. 3.0.4 [57], which employs a PCA to assess population genetic structure prior to outlier identification and is particularly suited to Pool-seq data [57]*.* PCADAPT was run with 5 replicates for our best *K* (*K* = 1 for the *leg pools and K* = 3 for the *population pools*) and only SNPs identified across all runs were considered to be candidates under selection.

Further information about the methods used can be found in Additional file 4.

## Results

### RAD-seq and mapping

Illumina sequencing yielded 9,250,492 reads for the four *population pools* (average 2,312,623 reads per pool) and 4,235,496 reads for the two *leg pools*. After filtering, we retained 8,232,334 reads for the *population pools* (average 2,058,084 reads per pool) and 3,602,671 reads for the two *leg pools* (see Additional file 5). De novo assembly in STACKS produced 76,168 RAD tags/loci, which we used as reference for mapping. Overall, we could map 6,345,433 reads (68.5%, x̄ = 1,586,358 reads per pool) for the *population pools* and 2,746,271 reads (64.8%, x̄ = 1,373,136 reads per pool) for the *leg pools* to our reference (see Additional file 5).

### Genome-wide variation and population genetic structure

In total we identified 29,721 SNPs that satisfied our filtering criteria in POPOOLATION2*.* The number of segregating sites (variable base positions in the genome) per population varied from 7362 to 9912 (x̄ = 8562, Table 2). Genetic diversity estimates, Watterson’s *θ* and Tajima’s *Π*, were extremely low for all populations, at *θ* = 0.0007 and *Π* = 0.0008 (Table 2). More stringent SNP filtering criteria only slightly increased Watterson’s *θ* and Tajima’s *Π* (see Additional file 6).

**Table 2:** Genetic diversity estimates for *Rediviva longimanus population pools* based on SNPs with a minor allele count of 2, minimum coverage of 10, maximum coverage ≤ 98%

Population	Number of segregating sites	Watterson’s *θ*	Tajima’s *Π*
AP	9912	0.0007	0.0008
LC	8196	0.0007	0.0008
LF	8779	0.0007	0.0008
LI	7362	0.0007	0.0008
Mean	8562	0.0007	0.0008

**Fig. 2:**
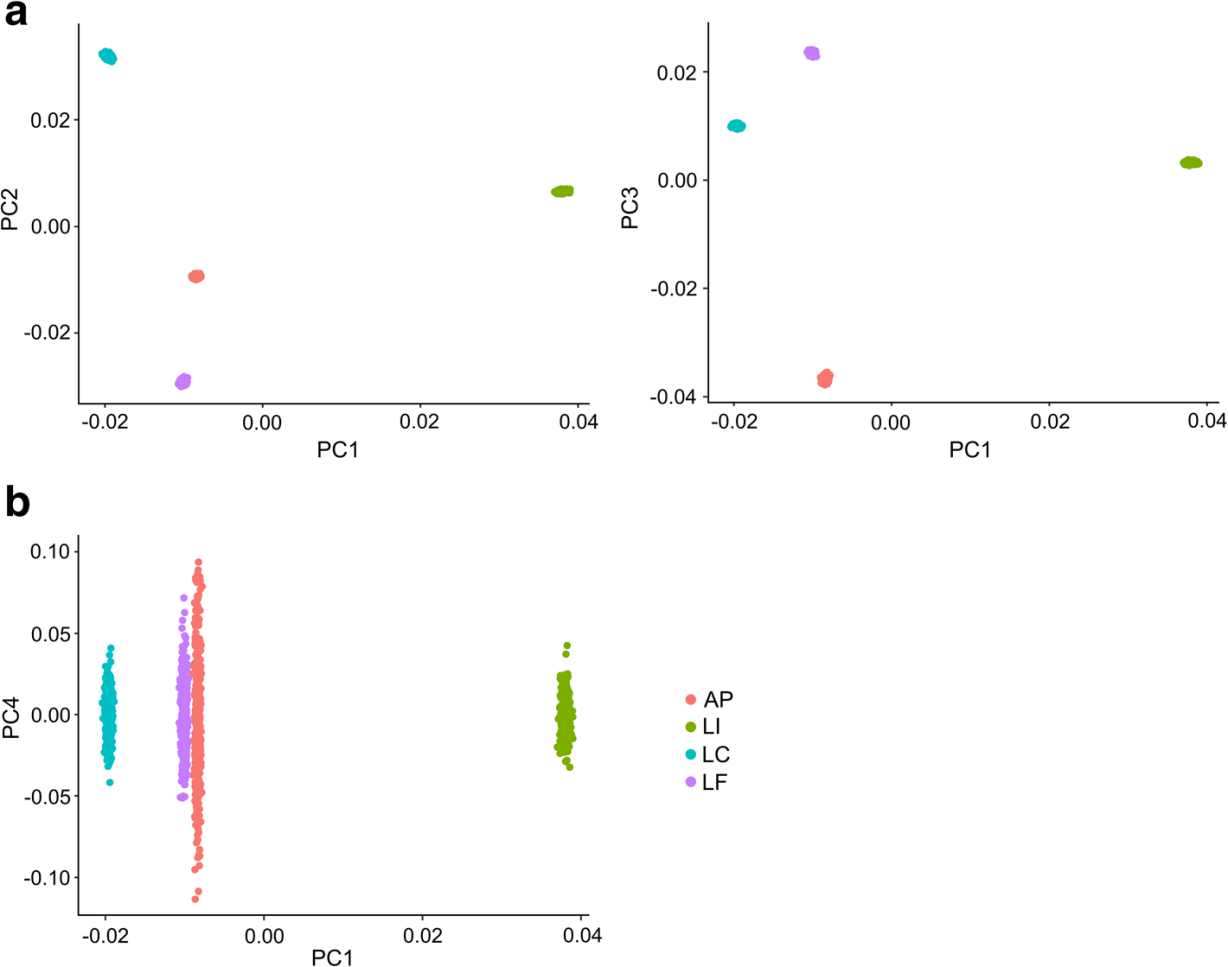
Population genetic structure of *Rediviva longimanus* according to principal component analysis (PCA). (**a**) PCA, performed in PCADAPT, suggested that three principal components explained most of the population genetic structure and revealed a clear separation into four population clusters. (**b**) Analysing more than three components did not contribute to disentangle further population genetic structure and revealed additional variance within rather than between clusters

Average *F*_ST_ values between population pairs were consistent and relatively high, ranging from 0.157 to 0.176 (mean *F*_ST_ across all populations = 0.165, lower 95% CI: 0.164, upper 95% CI: 0.167, Table 3), and even increased under more stringent filtering criteria: 0.219–0.241 (mean *F*_ST_ across all populations = 0.231, see Additional file 7). Excluding outlier loci did not markedly change *F*_ST_ estimates (mean *F*_ST_ across all populations = 0.164, lower 95% CI: 0.163, upper 95% CI: 0.166; see Additional file 7). Furthermore, PCA also supported the population genetic structure inferred by *F*_ST_ and clustered individuals according to their population of origin (Fig. 2).

**Table 3:** Geographic distances [km] (above diagonal) and pairwise *F*_ST_ values (below diagonal) at SNPs with a minimum count of the minor allele = 2, minimum coverage = 10, maximum coverage ≤ 98% for four *population pools* of *Rediviva longimanus*; lower and upper 95% CI’s of the *F*_ST_ values are given in brackets

	AP	LC	LF	LI
AP		76.39	66.24	99.53
LC	0.172 (0.169, 0.174)		66.40	92.82
LF	0.159 (0.157, 0.161)	0.157 (0.155, 0.160)		34.40
LI	0.176 (0.174, 0.179)	0.164 (0.161, 0.166)	0.163 (0.161, 0.166)

**Fig. 3:**
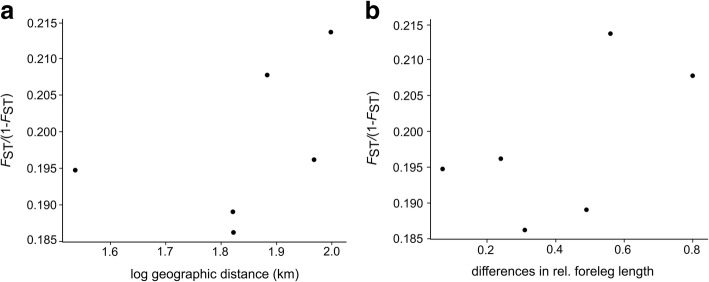
Regression of genetic differentiation of *Rediviva longimanus population pools* upon (**a**) foreleg length (FLL) and (**b**) geographic distance (log_10_). Although we failed to detect a significant pattern of isolation by adaptation (IBA) or isolation by distance (IBD) for the *R. longimanus* populations, there was a positive trend in both

We then tested if population genetic differentiation was correlated with population-level differences in mean FLL or rather with geographic distance. The relationship between genetic differentiation (as *F*_ST_/(1-*F*_ST_)) and log_10_ geographic distance was not significant (r^2^ = 0.21, *P* > 0.05, Fig. 3a). Genetic differentiation and differences in relative FLL were also not significantly correlated (r^2^ = 0.34, *P* > 0.05, Fig. 3b), although there was a positive trend in the relationship. Multiple matrix regression analysis including both geographic distances (log_10_) and relative FLL was also not significant (r^2^ = 0.36, *P* > 0.05).

Because non-significant results may arise through lack of statistical power, we estimated the power of our current analyses and the sample size needed to reject the null hypothesis of no IBD and IBA using the *R* package PWR v. 1.2.2 [64]. This analysis showed that, given our sample size, we needed an effect size of *r* = 0.90 to detect IBD and IBA. Our observed effects sizes (r) were clearly less than 0.90. Indeed, the statistical power of our analyses, given the observed effect sizes, was found to be low: 15% for IBD and 24% for IBA. Power analysis suggested that 10 or 8 populations would be needed to achieve power (1-β) of 0.80 to detect IBD or IBA, respectively.

### Demographic history of *Rediviva longimanus*

Demographic inference using FASTSIMCOAL2 suggested a bottleneck scenario to best fit our *population pools* (all four populations) since this scenario’s *AIC* value was smaller than those for the two alternative demographic models: constant *N*_*e*_ and population expansion (Table 4).

**Table 4:** Comparisons of three demographic models: bottleneck, constant size and population expansion, according to the Akaike Information Criterion (*AIC*) and Akaike’s weight of evidence (*w*)

Population	Model	*AIC*	*w*
AP			
	Bottleneck	180.643	1.00
	Constant	182.808	0.00
	Expansion	182.807	0.00
LC			
	Bottleneck	289.859	1.00
	Constant	308.174	0.00
	Expansion	388.800	0.00
LF			
	Bottleneck	255.909	1.00
	Constant	268.570	0.00
	Expansion	407.161	0.00
LI			
	Bottleneck	298.631	1.00
	Constant	317.317	0.00
	Expansion	352.877	0.00

### Genetic differentiation by FLL using *leg pools*

Genetic differentiation between the *leg pools* (*F*_ST_ = 0.296, lower 95% CI: 0.292, upper 95% CI: 0.299) was nearly twice as high as genetic differentiation among the *population pools* and dropped only slightly after excluding outlier loci (*F*_ST_ = 0.290, lower 95% CI: 0.291, upper 95% CI: 0.299). Hence, FLL might indeed play a non-negligible role in limiting gene flow (mating) between relatively long and relatively short-legged bees within and between populations.

### Outlier SNPs in the population pools and leg pools

**Fig. 4:**
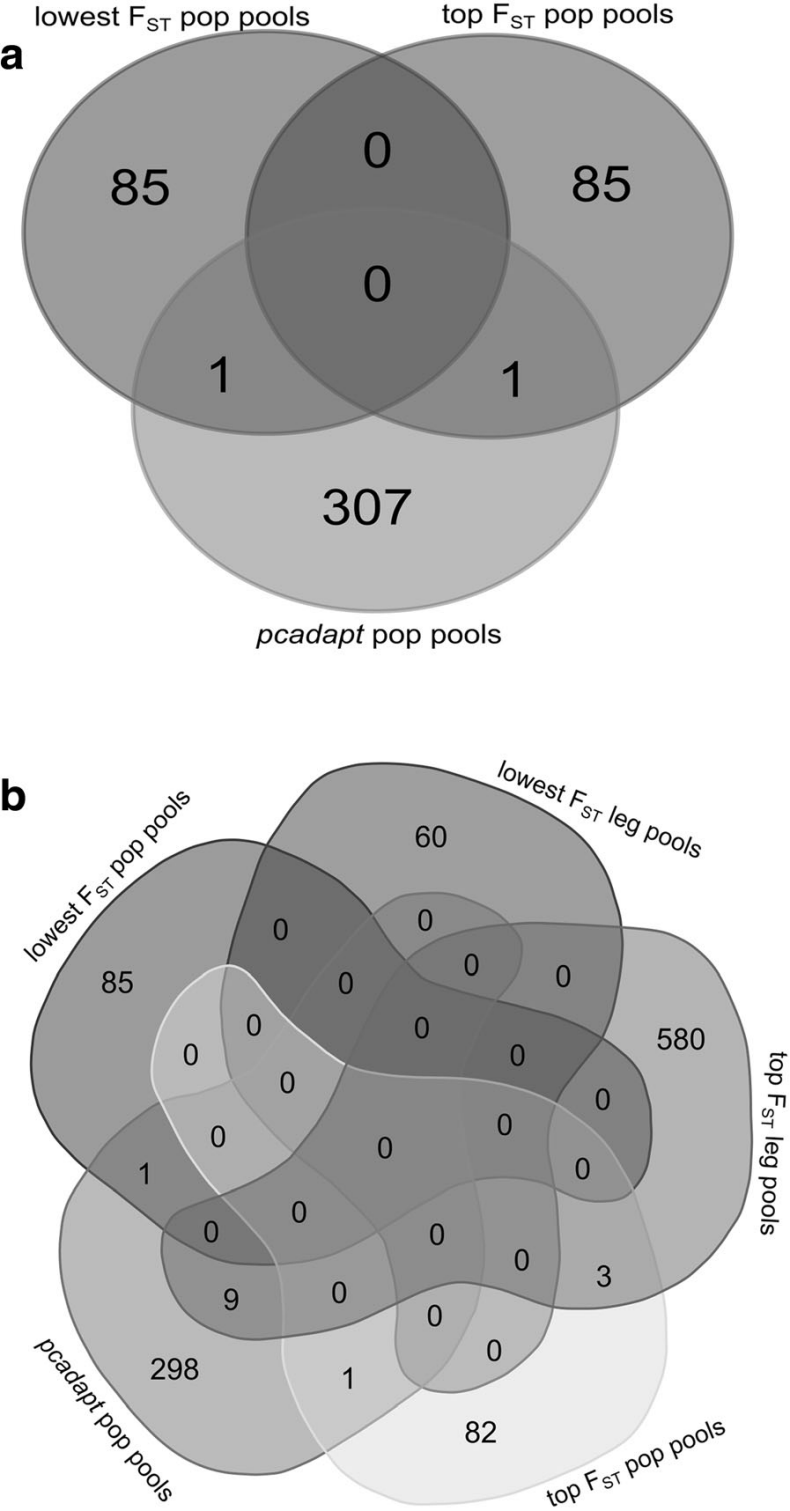
Overall we identified 1119 unique outlier RAD-tag loci. Shown is the overlap between (**a**) the different methods for the *population pool*s and between (**b**) the *population pools* and *leg pools* datasets. Diagrams were created with http://bioinformatics.psb.ugent.be/webtools/Venn

We identified 172 RAD-tags in the tails of the *F*_ST_ distribution for the *population pools* (0.5% threshold) and 652 in the *F*_ST_ tails for the *leg pool*s (5% threshold, see Table 5). PCADAPT analyses detected 326 candidate SNPs in 309 RAD-tags shared across all five runs for the *population pool*s, though only two of these also appeared in the tails of the *F*_ST_ distribution of the same dataset (Fig. 4a, Table 5). For the *leg pools*, PCADAPT did not identify any statistically significant outlier loci. Moreover, there was little overlap between the empirical *F*_ST_ outliers identified in the *leg pools* and the *population pools* (12 outliers overall, Fig. 4b). More detailed information on the outlier analyses can be found in Additional files 8 and 9.

**Table 5:** Tails of the *F*ST distributions in the *population pools* and *leg pools* datasets and number of outliers identified in those tails or by PCADAPAT. Note that we identified 1,133 outliers overall, with 14 shared between approaches or datasets, i.e. 1,119 unique outliers in total

Method	Dataset	*F*_ST_	Outlier RAD-tags
PCADAPT	*population pools*	-	309
*F*_ST_ (upper)	*population pools*	0.547 - 0.833	86
*F*_ST_ (lower)	*population pools*	0.001 - 0.014	86
PCADAPT	*leg pools*	-	-
*F*_ST_ (upper)	*leg pools*	1.000	592
*F*_ST_ (lower)	*leg pools*	0 - 0.002	60
**Total**			1133 (14 shared)

## Discussion

Our RAD-seq analyses of four *R. longimanus population pools* suggested marked genetic structuring of populations. Genetic differentiation between long-legged and short-legged bees pooled across populations was even more marked than population genetic differentiation, hinting at reduced gene flow based on leg length. All populations exhibited low genetic diversity estimates and seemed to have experienced bottleneck events, which is in accordance with field observations that *R. longimanus* populations are small and scattered.

### Genetic diversity, genetic structure and demographic history of *Rediviva longimanus* populations

Pronounced population genetic structuring and differentiation were detected between all population pairs of *R. longimanus*. Even over the relatively short geographic distance of 34 km, populations seemed to be highly differentiated (*F*_ST_ for LI versus LF: 0.163, lower 95% CI: 0.160, upper 95% CI: 0.166). For comparison, genetic differentiation of other ground-nesting wild bee species is often only significant over greater (> 50 km) geographic distances [65, 66].

Marked genetic differentiation over a small geographic distance might be a general feature of *Rediviva* bees and potentially of other flying insects in the South African winter rainfall area due to the region’s harsh climatic conditions [39]; the peak flowering season (August–September) is often cold and wet [67], which forces bees to forage under often unfavourable weather conditions. This might reduce bees’ foraging ranges and gene flow, resulting in increased genetic differentiation between populations [36, 39, 68]. In addition, *Rediviva* bees built their nests in the ground [25] and might have special nesting requirements. This might make the landscape appear fragmented to *Rediviva* bees and further reduce gene flow among their populations.

Low *N*_*e*_ could also account for the high population differentiation detected. A possibility is that bees are blown around by the harsh conditions of the Succulent Karoo, leading to high natal dispersal (i.e. near-panmixia), which, when coupled with very low *N*_*e*_, could generate a pattern of consistently high population genetic differentiation independent of geography. Though plausible, we consider this scenario unlikely as it cannot easily account for the consistent relationship between foreleg length and local *Diascia* floral spur length observed in *R. longimanus* (Hollens-Kuhr et al., unpublished observations) and other *Rediviva* spp. [28, 29].

Low *N*_*e*_ is, however, in agreement with personal observations of the rarity of the species (Kuhlmann, Hollens-Kuhr), and could also explain our low genetic diversity estimates for *R. longimanus*. Genetic diversity, measured as Watterson’s *θ* and Tajima’s *Π*, were at least one order of magnitude lower than estimates in other Pool-seq studies of non-threatened species [69, 70], though comparable to genetic diversity estimates for small island populations [71]. Our demographic analyses of *R. longimanus*, suggesting genetic bottlenecks, would also fit the low genetic diversity estimates inferred. During dry years, flower production can be very poor in the Succulent Karoo biome and most *Rediviva* species are likely to experience a marked reduction in population size, with some populations collapsing completely (Pauw, Kuhlmann, unpublished observations). Populations might thus frequently go through genetic bottlenecks, which would result in reduced genetic diversity, and which is probably only slowly restored once conditions become more favourable. The small, highly structured and potentially genetically depauperate *R. longimanus* populations are of conservation concern.

Pooling of individuals for genetic analysis might also have inflated our estimates of genetic differentiation, as suggested by Anderson et al. [72], who found that pools comprising few individuals might result in an artificial surplus of fixed loci. However, Anderson et al.’s conclusions were based on a very extreme example involving only six individuals per pool while our lowest pooled size (for population AP) contained 11 individuals. Thus, although Pool-seq might lead to biased allele frequency estimates and erroreous population genetic inferences when pool sizes and sequencing coverage are insufficient [72, 73], ours were likely adequate to ensure robust inference. First, our pool sizes were usually of the appropriate order of magnitude for accurate allele frequency estimates (≥ 25 individuals with a coverage of ≥20-30x), as suggested by Ferretti et al. [54]. Second, we followed highly stringent criteria in building our reference RAD-tags and in SNP calling (quality score ≥ 20, minimum coverage ≥ 10, minor allele frequency ≥ 2) to ensure accurate allele frequency estimation and reduce sequencing errors.

### Population genetic structure of *R. longimanus*, genetic drift and selection

We did not detect an association between population genetic differentiation in *R. longimanus* and geographic distance or variation in FLL, probably because we analysed only a relative small number of populations and thus lacked statistical power. *Rediviva longimanus* populations are small, thus the issue of statistical power is difficult to resolve. Reciprocal transplant experiment would help to assess if differences in FLL are locally adaptive [74]. But they are also difficult to implement on endemic and rare species that are of conservation concern, such as *R. longimanus*.

However, it is likely that genetic drift plays an important role in determining *R. longimanus* population genetic structure. First, populations are small in size (Kuhlmann, Hollens-Kuhr, unpublished observations), suggesting they are highly vulnerable to the effects of genetic drift. Moreover, we inferred population bottlenecks as the most likely demographic scenario for all populations studied. We also found low genetic diversity for all populations. Furthermore, populations seem to be significantly structured (average *F*_ST_ = 0.165), suggesting limited genetic exchange between populations, which would exacerbate the effects of drift.

Yet FLL might also have a non-negligible effect on genetic differentiation and result in reduced gene flow, as suggested by the high *F*_*ST*_ estimate between our *leg pools*. Though our *leg pools* dataset comprised individuals from different populations as well as with different leg lengths, the effect of population of origin per se on the *F*_ST_ estimate was probably slight. This is because we found all populations to exhibit a consistent, marked *F*_ST_ independent of geography and because we incorporated individuals from multiple – and often the same – populations into both the long and the short leg pools.

How variation in FLL per se translates into increased genetic differentiation, potentially because of reduced gene flow and limited mating between long and short legged morphs, is unknown. Sexual selection acting on FLL seems unlikely since variation in FLL is only displayed by females and, in bees, males are usually not the choosy sex but rather undertake scramble competition for mates [75]. Partial reproductive isolation is more likely due to habitat preference. It has already been shown that long legged *Rediviva* bees preferentially use long spurred flowers and vice versa [28, 34]. Long-legged bees might prefer localities with mainly long spurred plants while short-legged bees might preferentially occur at localities where short-spurred plants dominate, since bees will be more successful in extraction oil and hence provisioning offspring when they occur in localities with host plants possessing spur lengths that fit their FLL. Partial reproductive isolation due to habitat preference may then arise if mating takes place in the appropriate localities of daughters and their sons [7]. However, it is unknown where mating occurs in *R. longimanus*. Nevertheless, examples where local adaptation to a host plant increases genetic differentiation and may finally lead to reproductive isolation have been documented for several other insects, in particular phytophagous insects, e.g. the walking stick insect *Timema cristinae* [4, 9, 76], the leaf beetle *Neochlamisus bebbianae* [5] or the apple maggot fly *Rhagoletis pomonella*, [19, 20]; see also [77] for a more complete list.

### Candidate genes under selection

Overall, we detected 1119 outlier loci, though there was little consistency in outlier loci identified by the different methods and datasets. Our pooled RAD-seq approach analysed only a small proportion of the *R. longimanus* genome and thus likely missed important genes underpinning local adaptation. Whole genome sequencing rather than our RAD-seq approach would be more powerful to address the genetics of local adaptation and to identify candidate loci underlying FLL, which we assume to be locally adaptive and experience strong selection.

We note, though, that selection may not always favour bees with the longest legs but rather may favour those with the best fitting legs. Simulations suggest that bees with legs much longer than the floral spurs of their hosts are unable to successfully collect oil [6]. Selection, even if strong, may then maintain multiple alleles, namely for both long and for short legs, in the same population.

## Conclusions

We found pronounced genetic differentiation among *R. longimanus* populations and low genetic diversity, likely because of low *Ne* and limited dispersal, compounded by recent bottleneck events. Genetic drift seemed to be important in structuring *R. longimanus* populations, but FLL might also reduce gene flow, as indicated by high genetic differentiation between our *leg pools*. Future studies including additional populations are required to test if neutral evolutionary processes such as genetic drift and migration or host plant adaptation are more important in structuring *R. longimanus* populations and whether FLL is associated with reduced gene flow and reproductive isolation. Nevertheless, our study is a first step to understand better the population genomics of an important pollinator in the Succulent Karoo biodiversity hotspot.

## Additional files

Additional file 1: Pairwise test for significant differences in relative FLL between *Rediviva longimanus population pools*. (XLSX 10 kb)

Additional file 2: Picture showing the differences in FLL between *population pool* LC (Keiskie Mountains) and LF (Farm Papkuilsfontain). (PDF 180 kb)

Additional file 3: Locations and number of samples per location for the two *Rediviva longimanus leg pools*. Mean FLL and standard deviations for the two *leg pools* are also indicated. (XLSX 10 kb)

Additional file 4: Additional methods. (DOCX 110 kb)

Additional file 5: Summary statistics for the number of reads sequenced, reads retained after filtering and reads successfully mapped to the consensus reference sequence for the *Rediviva longimanus population pools* and *leg pools*. (XLSX 10 kb)

Additional file 6: Genetic diversity estimates for the *Rediviva longimanus population pools* including only SNPs with a minor allele count of 6, minimum coverage of 20 and maximum coverage ≤ 98%. (XLSX 11 kb)

Additional file 7: Pairwise *F*_*ST*_ for the *Rediviva longimanus population pools* after excluding loci potentially under selection (above diagonal) and *F*_*ST*_ (below diagonal) under more stringent SNP filtering criteria (minimum count of the minor allele = 6, minimum coverage = 20, maximum coverage ≤ 98%). (XLSX 12 kb)

Additional file 8: Additional results for the outlier analysis, in particular for outlier annotation and GO enrichment. (DOCX 325 kb)

Additional file 9: BLAST and GO annotation of all 136 outliers identified for the *Rediviva longimanus population* and *leg pool* data. The data set and methods with which the outliers were identified are also given. (XLSX 61 kb)
