# Supplementary material for: Small and genetically highly structured populations in a long-legged bee, Rediviva longimanus, as inferred by pooled RAD-seq
Source: BMC Evol Biol. 2018 Dec 19;18:196. doi: 10.1186/s12862-018-1313-z (PMC6300007; doi:10.1186/s12862-018-1313-z)
Supplement: Supplementary file 2 — Picture showing the differences in FLL between population pool LC (Keiskie Mountains) and LF (Farm Papkuilsfontain). (PDF 180 kb) [file 12862_2018_1313_MOESM2_ESM.pdf]

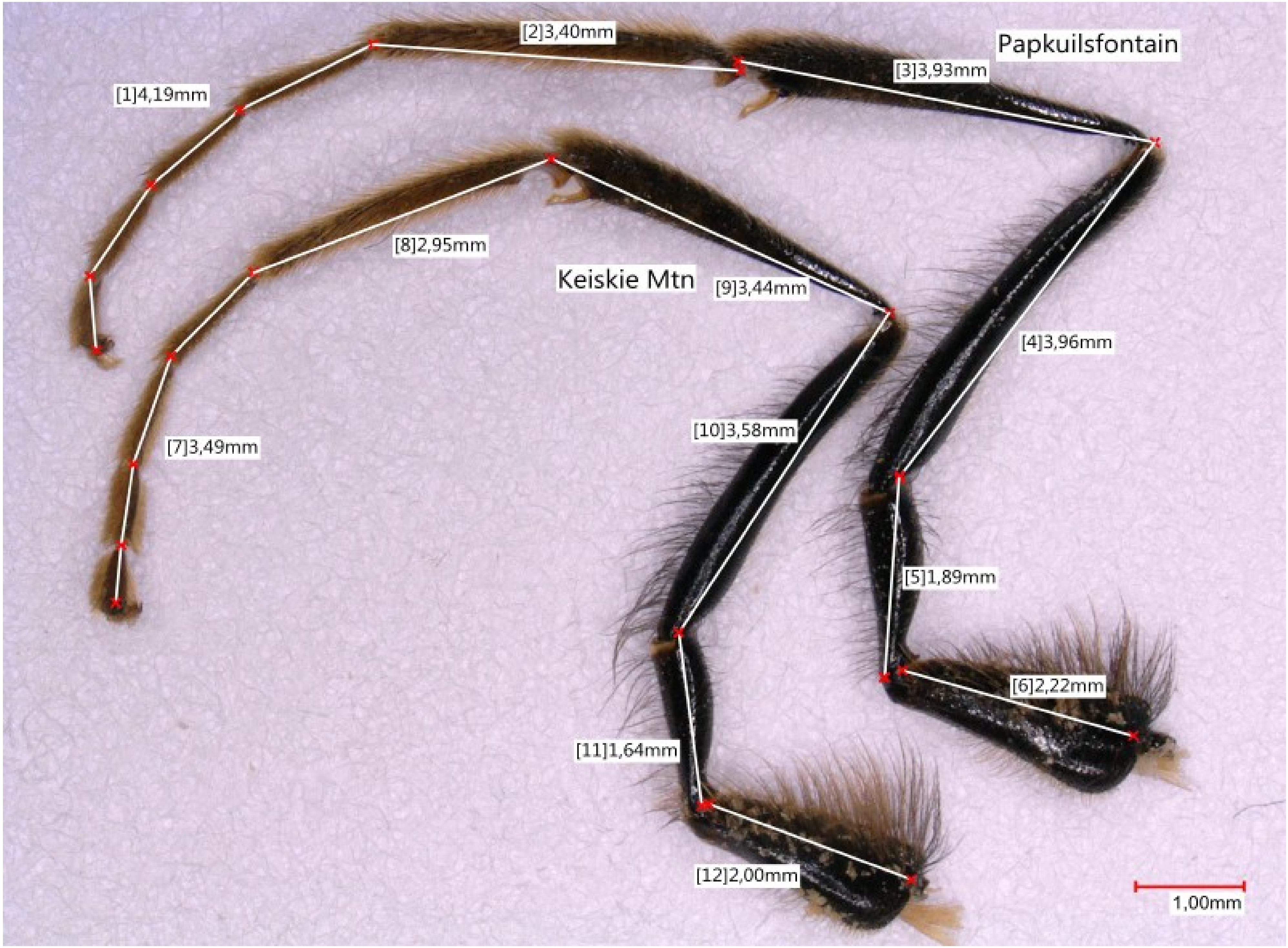

Additional file 2: Picture showing the differences in FLL between *population pool* LC (Keiskie Mountains) and LF (Farm Papkuilsfontain)
