# Supplementary material for: Small and genetically highly structured populations in a long-legged bee, Rediviva longimanus, as inferred by pooled RAD-seq
Source: BMC Evol Biol. 2018 Dec 19;18:196. doi: 10.1186/s12862-018-1313-z (PMC6300007; doi:10.1186/s12862-018-1313-z)
Supplement: Supplementary file 4 — Additional methods. (DOCX 110 kb) [file 12862_2018_1313_MOESM4_ESM.docx]

**Additional File 4: Additional methods**

**Study design**

In our study, we prepared pools of *R. longimanus* individuals according two different schemes:

1. *population pools*, where we pooled individuals according to the sampling site
2. *leg pools*, where we pooled individuals according to relative leg length

While the *population pools* are suitable to address the genetic structure of *R. longimanus* populations and potentially also to identify candidate outlier loci related to FLL due to significant inter-population variation in mean FLL, the *leg pool* data set is designed with the intention to test for reproductive isolation between FLL variants (long *versus* short) and to detect outliers associated with FLL. Thus, by pooling according to FLL, we aimed to generate pools that showed maximal morphological differences between pools but minimal differences within a pool. Pooling individuals from different populations but with the same morphology, as in our two *leg pools*, has been recognised as a valid and highly useful approach for identifying candidate genes for host adaptation [1,2].

For FLL analyses we used the relative foreleg length, i.e. standardized by body size, rather than absolute length in order to control for length variation due to total body size. We measured the foretarsus length, as a proxy for foreleg length, divided by the head width, as a proxy for body size. Head width is a frequently used estimate for body size as both have been shown to be highly correlated in several insects, including *Rediviva* [8]. Foretarsus length is a reliable proxy of foreleg length because, for some *Rediviva* species that use *Diascia* as floral hosts, elongation of the front leg is mainly achieved by an increase in length of the foretarsus [9].

**Data processing and SNP calling**

An initial quality control of the RAD-seq reads using FASTQC v. 0.11.5 (http://www.bioinformatics.babraham.ac.uk/projects/fastqc) revealed overall high sequence quality. Subsequently, sequence reads were demultiplexed, filtered for quality and trimmed of 10 bp MID sequences using the *process_radtags* script of STACKS v. 1.42 [47] under default settings. Since there is no reference genome available for *R. longimanus* or a close relative, we identified RAD loci *de novo* using *denovo_map.pl* in STACKS. We ran the script by requiring a stack depth of at least five reads (m = 5), allowing two mismatches between loci for a single individual (M = 2) and no mismatch when building the catalogue loci (n = 0). Moreover, we enabled both the removal (-r) and deleveraging (-d) algorithms to remove highly-repetitive stacks and resolve over-merged tags, respectively. After running the core pipeline, we employed RXSTACKS to remove loci with a log-likelihood below -20 and confounding loci, i.e. multiple genomic loci matching a single catalogue locus, and then re-ran the CSTACKS and SSTACKS scripts individually to obtain a filtered loci catalogue.

In STACKS we also tested several parameter combinations of *m*, *M* and *n* (results not shown). While varying *m* and *M* slightly affected the number of stacks and markers, varying *n* from 1 to 3 did not change the number of markers obtained and we thus believe that under-merging loci is not particularly problematic for our data. Moreover, recent work has shown that, in most cases, alternative STACKS parameter settings do not affect population genetic inferences [3]. Thus, for all subsequent analyses, we only used the STACKS output for our optimal parameter setting, i.e. the setting yielding the highest number of SNPs while simultaneously avoiding under-or over-merging of locus stacks.

Since STACKS was not specifically designed for the use of pooled samples and its SNP calling algorithm is therefore likely to miss low frequency variants in the pool, we used POPOOLATION2 [48] for SNP calling. We mapped all our RAD reads against the reference catalogue created in STACKS using the *bwa mem* algorithm of BWA v. 0.7.12 [49] as this is superior to alternative mapping algorithms for pooled data [50]. Mapping results were filtered for a minimum Phred quality score of 20 and converted into mpileup format in SAMTOOLS v. 0.1.19 [51]. Finally, we synchronized allele frequencies of all populations in the mpileup file using *mpileup2sync.jar* in POPOOLATION2. For each population pair, we then calculated the allele frequency difference at each position with a minimum coverage of ten and a minimum minor allele count of two using the *snp-frequency-diff.pl* script of POPOOLATION2. We then exported the SNPs identified by POPOOLATION2 into GENEPOP format using the *subsample_sync2GenePop.pl* script and a custom perl script kindly provided by Dr. B. Guo (University of Helsinki) by randomly sampling bases to obtain a uniform coverage of 10x for each SNP per population.

**Genome-wide variation and population genetic structure**

In addition to *F*_ST_, we also assessed population genetic structure by principal component analyses (PCA) in the R package PCADAPT v. 3.0.4 [4]*.* The number of principle components, *K*, that are most appropriate to describe population genetic structure were inferred based on the scree plot output of PCADAPT, which shows the eigenvalues of the covariance matrix in descending order. Up to a constant value, the eigenvalues are proportional to the proportion of variance explained by each principal component. According to Cattell’s rule [5], the components, *K*, that correspond to eigenvalues before a constant value is reached are most appropriate to describe population genetic structure.

**Demographic history of *Rediviva longimanus***

Since estimates for Watterson’s *θ* and Tajima`s *Π* suggested very low genomic diversity for all *population pools*, we tested for a bottleneck in each population using FASTSIMCOAL2 v. 2.5.2.21 [6]. FASTSIMCOAL2 employs coalescence simulations to generate the site frequency spectrum (SFS) expected under a given set of demographic parameters and computes their (composite) likelihood [6].

For demographic inferences, we first excluded RAD tags with SNPs potentially under selection (see below) using a custom bash script and then inferred the folded SFS in POOL-HMM v. 1.4.3 [7] based on the minor allele frequencies computed with POPOOLATION2. An SFS for each population was computed using similar criteria as in previous analyses, i.e. a minimum coverage of 10, a quality threshold of 20, exclusion of the top 2% covered regions, and other parameters left as default. Probability outputs of POOL-HMM were multiplied by the number of positions analysed to obtain count data used by FASTSIMCOAL2.

We chose to model every population individually since PCA and *F*_ST_-analyses suggested marked population genetic structure. In FASTSIMCOAL2 we first estimated model parameters using sequential Markov coalescence simulations and a conditional maximization algorithm (ECM), where each parameter of the model is maximized in turn [6]. The ECM procedure was run through 40 cycles, for which each composite-likelihood was calculated using 100,000 simulations. The criterion to stop the ECM cycles was defined as a minimum relative difference in parameters between two iterations of 0.001. To avoid oversampling of parameter values at local maxima across the composite likelihood surface, we ran 50 replicates, each with different starting conditions, using a custom bash script. Finally, we chose the replicate with the highest estimated maximum likelihood score. In addition to a bottleneck scenario, we also modelled a constant population size scenario and a population expansion scenario using the same procedure and simulation settings as described above. Model comparisons were performed according to the Akaike Information Criterion (*AIC*) and Akaike’s weight of evidence (*w*) in favour of the *i*-th model over all models, as suggested by Excoffier et al. [6]:

$$wi=\frac{exp(-0.5\Delta i)}{\sum_{r=i}^{R} exp(-0.5\Delta r)}$$

where Δi = AIC_i_ - AIC_min_ and Δr the difference in the AIC for each pairwise model comparison.

**Outlier SNP detection in the *population pool* data set**

Since mean FLL was significantly different for most population pairs (see Additional file 1), we tested for signals of selection at loci associated with FLL in our *population pools* using two approaches which suit our pooled data sets better than conventional outlier detection approaches [10–12]. Indeed, test runs with these conventional methods revealed that they were not suitable for pooled data (LOSITAN) or that they required a larger sample size for robust inference (BAYESCAN, BAYESCENV). In the first outlier detection approach used, we extracted loci in the 0.5% tails of the *F*_ST_ distribution [as in 13] as calculated in POPOOLATION2. We considered the loci with the highest *F*_ST_ values (upper 0.5% tail) as candidates for divergent selection and the loci with the lowest *F*_ST_ (lower 0.5% tail) as candidates for balancing selection, in accordance with the rationale underlying *F*_ST_-outlier detection tools such as BAYESCAN [11] or LOSITAN [10].

Secondly, we used PCADAPT v. 3.0.4 [4], which addresses the caveats of the *F*_ST_ approaches of BAYESCAN by employing a PCA to assess population genetic structure prior to outlier identification and is particularly suitable for Pool-seq data [4]*.* Population genetic structure is assessed via PCA to determine the *K* components that best describe structure and selection is then inferred with the Mahalanobis distance. Candidates under selection (directional or balancing) are SNPs for which the vector of z-scores describing the relation between a SNP and the *K* principal components does not follow the distribution of the main bulk of points, which correspond instead to SNP markers excessively related to population genetic structure.

For outlier detection in PCADAPT v. 3.0.4, we first generated a genotype matrix based on the read count data by sampling 500 individuals from the *population pool*. Since the scree plot suggested *K* = 3 best describes population genetic structure (see Fig. 1 below), we computed the PCADAPT test statistic for *K* = 3, using default settings, a minor allele frequency of 0.10 and a false discovery rate (FDR) of 0.01. PCADAPT was then run with 5 replicates for our best *K* = 3 and only SNPs identified across all runs were considered to be candidates under selection. In order to determine which of the outliers identified are highly associated with foreleg length, we regressed PCADAPT outliers upon relative foreleg length in R (function: lm).


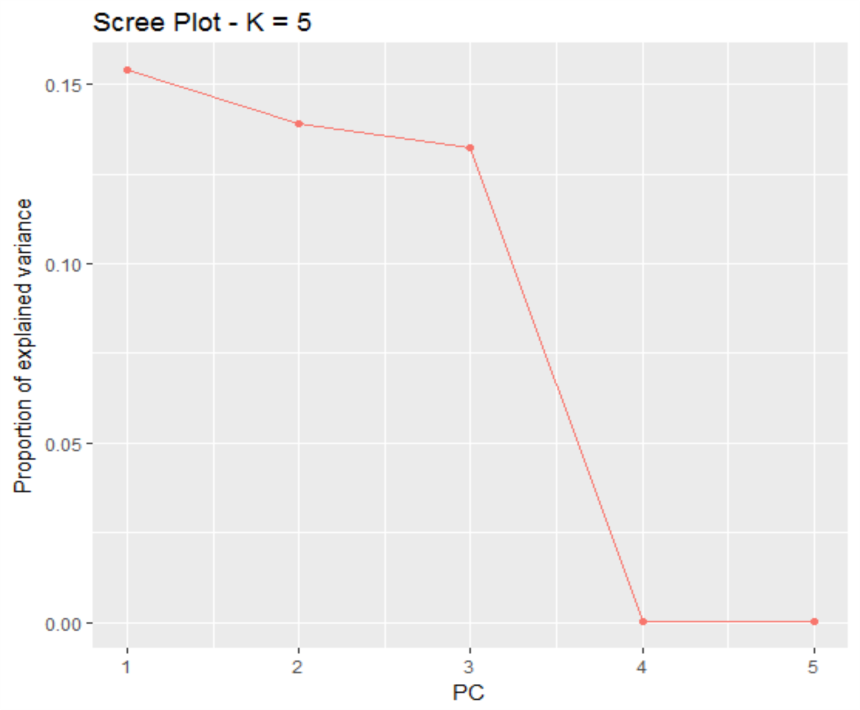


**Fig. 1.** Scree plot used to infer the most suitable K to describe population genetic structure of the *Rediviva longimanus population pools.* (PDF)

**Outlier SNP detection in the *leg pool* data set**

We used the *leg pool* data set to identify outliers associated with differences in foreleg length, employing the same outlier identification approaches as for the *population pool* data set. We considered the following loci as candidates under selection i) loci with an *F*_ST_ of 1, corresponding to the upper 5% tail of the *F*_ST_ distribution (i.e. divergent selection) and in the lower 5% tail of the *F*_ST_ distribution (i.e. balancing selection), in POPOOLATION2 and ii) outliers identified in PCADAPT [4]*.* PCADAPT settings were identical to those used for the *population pool* data set apart from the fact that we ran PCADAPT under *K* = 1 as one population was inferred to best describe genetic structure for the *leg pools*.

**SNP outlier annotation**

RAD-tag sequences including outlier SNPs inferred with the approaches used above (PCADAPT*,* empirical *F*_ST_ outliers) were annotated using BLAST2GO [14]. BLAST2GO was run under default parameters (word size 3, HSP length cut-off 33) using a *blastx* search against nucleotide entries for bees (Apoidea) only and only considering hits with an e-value ≤ 1.0E-5. We allowed a maximum of four BLAST hits per RAD-tag locus and only annotated sequences with all four hits to the same homologous gene in different taxa, or when the best hit was significantly better than the three alternative hits. Functional annotation and gene ontology (GO) term mapping were also performed in BLAST2GO under default parameters and manually supplemented with GO terms from the *Bombus terrestris* and *Bombus impatiens* genome annotations (http://seth.barribeau.com/home/news/). Visualisation of GO annotations was carried out with the WEGO software [15]. Furthermore, we performed GO term enrichment analysis of our 126 reliably annotated outliers with all annotated RAD tags as the reference data set using a two-tailed Fisher’s exact test and default settings (FDR= 0.05) in BLAST2GO.

**References**

1. Egan SP, Nosil P, Funk DJ. Selection and genomic differentiation during ecological speciation: isolating the contributions of host association via a comparative genome scan of Neochlamisus bebbianae leaf beetles. Evolution (N Y). 2008;62:1162–81.

2. Nosil P, Egan SP, Funk DJ. Heterogeneous genomic differentiation between walking-stick ecotypes: “Isolation by adaptation” and multiple roles for divergent selection. Evolution (N Y). 2007;62:316–36.

3. Rodríguez-Ezpeleta N, Bradbury IR, Mendibil I, Álvarez P, Cotano U, Irigoien X. Population structure of Atlantic mackerel inferred from RAD-seq-derived SNP markers: effects of sequence clustering parameters and hierarchical SNP selection. Mol Ecol Resour. 2016;16:991–1001.

4. Luu K, Bazin E, Blum MGB. pcadapt: An R package to perform genome scans for selection based on principal component analysis. Mol Ecol Resour. 2017;17:67–77.

5. Cattell RB. The scree test for the number of factors. Multivariate Behav Res. 1966;1:245–76.

6. Excoffier L, Dupanloup I, Huerta-Sánchez E, Sousa VC, Foll M. Robust demographic inference from genomic and SNP data. PLoS Genet. 2013;9:e1003905.

7. Boitard S, Kofler R, Françoise P, Robelin D, Schlötterer C, Futschik A. Pool-hmm: A Python program for estimating the allele frequency spectrum and detecting selective sweeps from next generation sequencing of pooled samples. Mol Ecol Resour. 2013;13:337–40.

8. Steiner KE, Whitehead VB. Pollinator adaptation to oil-secreting flowers-*Rediviva* and *Diascia*. Evolution (N Y). 1990;44:1701–7.

9. Steiner KE, Whitehead VB. Oil flowers and oil bees: further evidence for pollinator adaptation. Evolution (N Y). 1991;45:1493–501.

10. Antao T, Lopes A, Lopes RJ, Beja-Pereira A, Luikart G. LOSITAN: A workbench to detect molecular adaptation based on a F st -outlier method. BMC Bioinformatics [Internet]. 2008;9:1–5. Available from: http://dx.doi.org/10.1186/1471-2105-9-323

11. Foll M, Gaggiotti O. A genome-scan method to identify selected loci appropriate for both dominant and codominant markers: A Bayesian perspective. Genetics. 2008;180:977–93.

12. de Villemereuil P, Gaggiotti OE. A new F_ST_-based method to uncover local adaptation using environmental variables. Methods Ecol Evol. 2015;6:1248–58.

13. Fabian DK, Kapun M, Nolte V, Kofler R, Paul S, Schlo C. Genome-wide patterns of latitudinal differentiation among populations of *Drosophila melanogaster* from North America. Mol Ecol. 2012;21:4748–69.

14. Conesa A, Götz S, García-Gómez JM, Terol J, Talón M, Robles M. Blast2GO: A universal tool for annotation, visualization and analysis in functional genomics research. Bioinformatics. 2005;21:3674–6.

15. Ye J, Fang L, Zheng H, Zhang Y, Chen J, Zhang Z, et al. WEGO : a web tool for plotting GO annotations. Nucleic Acids Res. 2006;34:W293–7.
