# Supplementary material for: Small and genetically highly structured populations in a long-legged bee, Rediviva longimanus, as inferred by pooled RAD-seq
Source: BMC Evol Biol. 2018 Dec 19;18:196. doi: 10.1186/s12862-018-1313-z (PMC6300007; doi:10.1186/s12862-018-1313-z)
Supplement: Supplementary file 8 — Additional results for the outlier analysis, in particular for outlier annotation and GO enrichment. (DOCX 325 kb) [file 12862_2018_1313_MOESM8_ESM.docx]

**Additional File 8: Additional results**

**Outlier annotation**

From the 1,119 unique outliers identified with either PCADAPT or from the empirical *F*_ST_ distribution approach, we obtained a blast hit for 126 outlier RAD-tags (12%) in both pool data sets (*population pools* and *leg* pools; see Table 1 below). Among these 126 outliers we also identified candidate loci for FLL regulation such as the Hox gene *Sex combs reduced* (*Scr*), 12 other transcription factors and five genes involved in the regulation of transcription or cell division/DNA replication (see Additional file 10).

In most insect taxa the expression of *Scr* is needed to suppress wing development and to establish the identity of legs in the first thoracic segment [73–76]. But so far no role in leg length regulation has been suggested for *Scr*. The *Scr* gene is a promising starting point for understanding the genetic architecture of FLL variation. However, the *Scr* outlier was only detected in the lower tail of the *F_ST_* distribution in *leg pools*; we could not confirm it using PCADAPT or the *population pool* data. Future studies empolying individual SNP genotyping of the *Scr* locus and functional studies of bees in earlier developmental stages (e.g. pupal stages) would be required to support the role of the *Scr* locus in FLL regulation. Moreover, as our pooled RAD-seq design sampled only a small proportion of the *R. longimanus* genome, our approach might have missed other important regulators of FLL variation, which can only be tackled with whole genome sequencing.

**Table 1.** Number of outliers identified in PCADAPAT or the tails of the *F*_ST_ distribution in the *population* and *leg pool* dataset that yielded a BLAST hit. Note that we identified 1,133 outliers overall with 14 shared between approaches or datasets, i.e. 1,119 unique outliers overall.

| **Method** | **Dataset** | ***F*_ST_** | **Outlier RAD-tags identified** | **Outliers blasted** |
| --- | --- | --- | --- | --- |
| PCADAPT | *population pools* | - | 309 | 34 |
| *F*_ST_ (upper) | *population pools* | 0.547 - 0.833 | 86 | 7 |
| *F*_ST_ (lower) | *population pools* | 0.001 - 0.014 | 86 | 24 |
| PCADAPT | *leg pools* | - | - | - |
| *F*_ST_ (upper) | *leg pools* | 1 | 592 | 53 |
| *F*_ST_ (lower) | *leg pools* | 0 - 0.002 | 60 | 7 |
| **Total** |  |  | 1,133 (14 shared) | 126 |

From all the outliers identified that we were able to annotate (126 overall), 62 could be associated with GO terms, which tested for GO term enrichment. GO annotation revealed several loci that might be promising candidates for controlling processes such as limb growth. For example, the “molecular function” of many outliers related to either catalytic activity (31.3 %) or binding processes (43.8%), in particular to nucleic acids or nucleotides (Fig. 1). The GO ‘ion binding’ was also found to be significantly overrepresented in GO enrichment analyses (Fig. 2). Some outliers also have regulatory activity and control protein translation (2.1%) or act as molecular transducer (4.2%, Fig. 1).


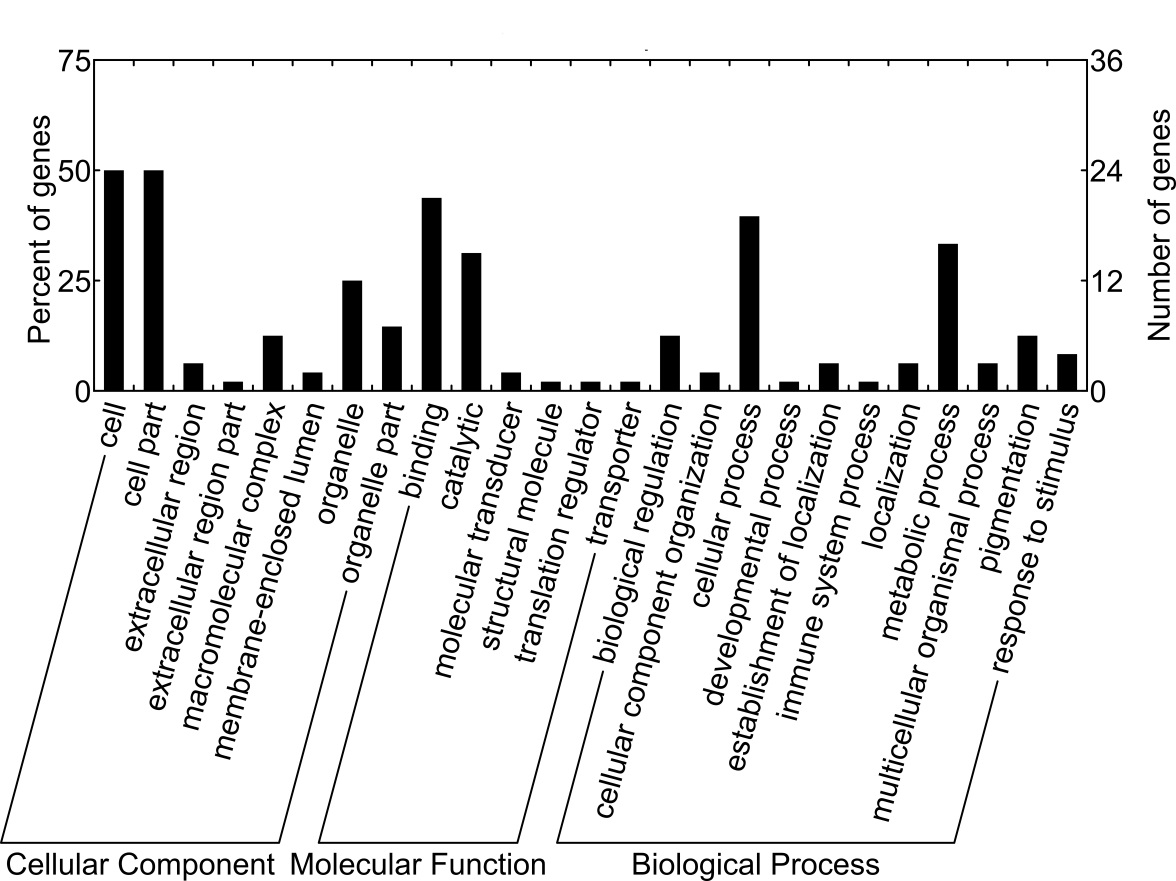


**Fig. 1** Gene ontology (GO) classification of the 62 outlier loci we successfully blasted and annotated with BLAST2GO*.* Plotted are the number and percentage of outliers falling into the three main ontologies (cellular component, molecular function and biological process)


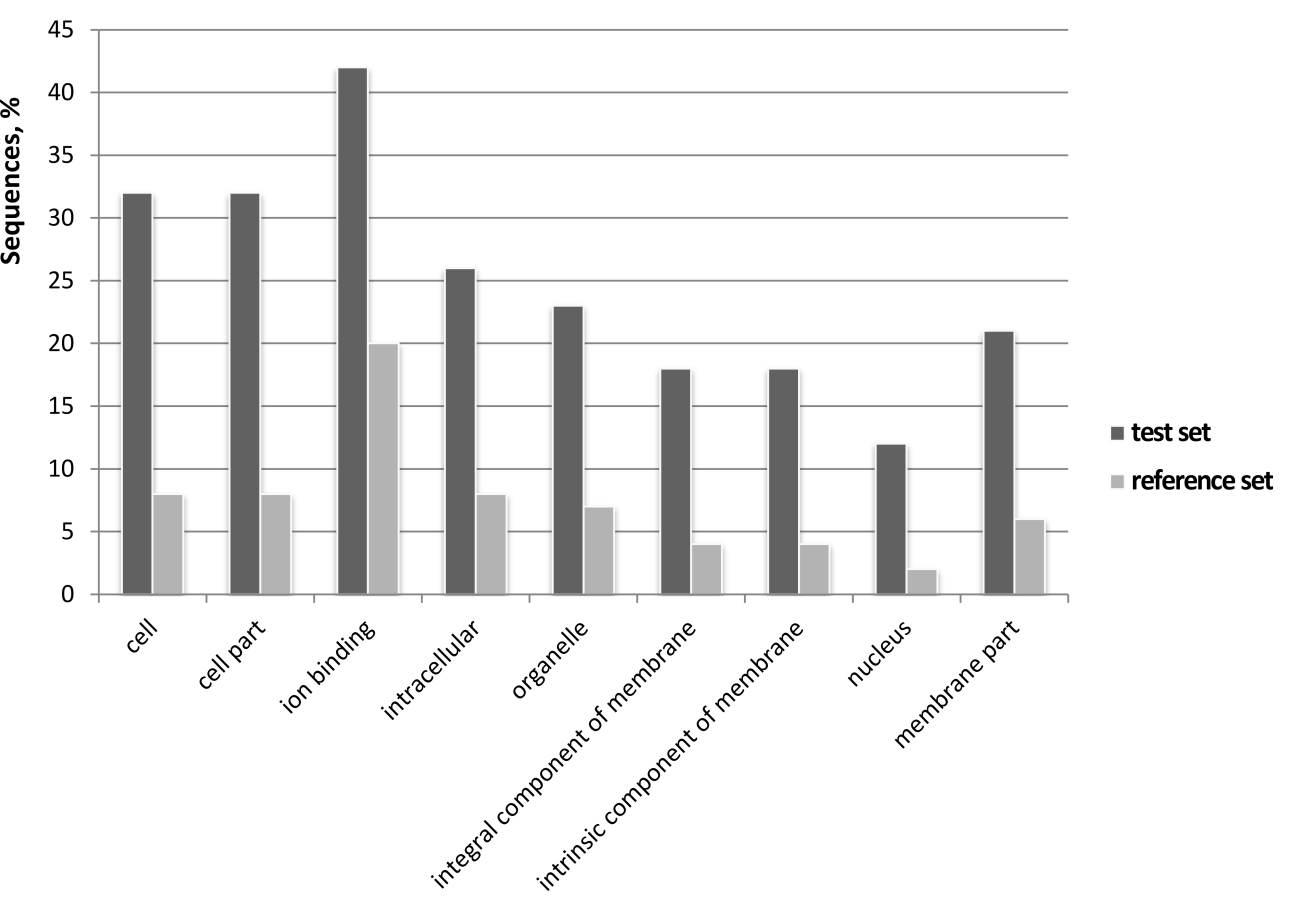


**Fig. 2** GO categories that were found to be significantly overrepresented in our outlier (test) set compared to the reference during GO term enrichment analyses
